# Dual-Slope Diffuse Reflectance Instrument for Calibration-Free Broadband Spectroscopy

**DOI:** 10.3390/app11041757

**Published:** 2021-02-16

**Authors:** Giles Blaney, Ryan Donaldson, Samee Mushtak, Han Nguyen, Lydia Vignale, Cristianne Fernandez, Thao Pham, Angelo Sassaroli, Sergio Fantini

**Affiliations:** Department of Biomedical Engineering, Tufts University, Medford, MA 02155, USA

**Keywords:** broadband diffuse reflectance spectroscopy, frequency-domain near-infrared spectroscopy, dual-slope, absorption spectra

## Abstract

This work presents the design and validation of an instrument for dual-slope broadband diffuse reflectance spectroscopy. This instrument affords calibration-free, continuous-wave measurements of broadband absorbance of optically diffusive media, which may be translated into absolute absorption spectra by adding frequency-domain measurements of scattering at two wavelengths. An experiment on a strongly scattering liquid phantom (milk, water, dyes) confirms the instrument’s ability to correctly identify spectral features and measure absolute absorption. This is done by sequentially adding three dyes, each featuring a distinct spectral absorption, to the milk/water phantom. After each dye addition, the absorption spectrum is measured, and it is found to reproduce the spectral features of the added dye. Additionally, the absorption spectrum is compared to the absorption values measured with a commercial frequency-domain instrument at two wavelengths. The measured absorption of the milk/water phantom quantitatively agrees with the known water absorption spectrum (*R*^2^ = 0.98), and the measured absorption of the milk/water/dyes phantom quantitatively agrees with the absorption measured with the frequency-domain instrument in six of eight cases. Additionally, the measured absorption spectrum correctly recovers the concentration of one dye, black India ink, for which we could accurately determine the extinction spectrum (i.e., the specific absorption per unit concentration). The instrumental methods presented in this work can find applications in quantitative spectroscopy of optically diffusive media, and particularly in near-infrared spectroscopy of biological tissue.

## 1. Introduction

Diffuse optics is concerned with the propagation of light in highly-scattering or diffusive media. One notable application is Near-Infrared Spectroscopy (NIRS) of biological tissue, which is typically performed in the wavelength range from about 600 nm to about 1000 nm [1–3]. Diffuse optics finds a variety of applications in several fields of study. In food science [4], it may be applied for inspection [5] or evaluation [6]. In pharmaceutical manufacturing, diffuse optics may join other process analytical technologies, for example to analyze and characterize particles [7] or powders [8]. A few other examples include archaeological soil analysis [9], dendrology (study of wood) [10], and art authentication analysis [11]. In the study of biological tissue, diffuse optics finds applications in basic research, medical diagnostics, and physiological monitoring. Examples include clinical brain monitoring [12], the study of brain activation [13], breast imaging [14], and muscle measurements in sports science [15]. This is by no means an exhaustive list. The work that we present in this article aims to improve the accuracy and robustness of quantitative broadband spectroscopy of optically turbid media, and it is relevant to a variety of applications of diffuse optics, even though we mostly focus on its role in the area of biomedical optics.

Quantitatively, light propagation in diffusive media is characterized by a lower probability of absorption (related to the absorption coefficient; *μ*_*a*_) compared to the probability of effectively isotropic scattering (related to the reduced scattering coefficient; $\mu_{s}^{\prime }$). These two optical properties are the chief quantities of interest in the field of diffuse optics and diffuse biomedical optics [2,3,16,17], and they feature a wavelength dependence that is often of crucial importance. In the case of most biological tissues, the dominating scattering condition of diffuse optics is realized in the NIR optical window. Measurements of the wavelength dependent absorption of tissue yields information about the concentration of chromophores with known extinction spectra, while measurements of scattering spectra yields structural information related to the size and density of scattering centers [2].

These properties are often obtained in the reflectance geometry, where light is delivered onto the sample surface and detected at some distance away from the source on the same surface. The primary difficulty in these measurements is the decoupling of absorption and scattering contributions to the measured optical signal. One method to achieve this is by using a source light with a temporally varying intensity. These time-resolved methods fall into two categories: time-domain methods where the light emission has an impulse profile, and Frequency-Domain (FD) methods where the light emission has a sinusoidal profile [1,18]. Focusing on FD-NIRS, the measurement of both absorption and scattering can be achieved via a calibrated measurement of the spatial dependence (as a function of the distance from the source on the tissue surface) of the amplitude and phase of the detected modulated intensity (which may be represented as a complex reflectance) [19–21].

This spatially dependent measurement of FD-NIRS data to achieve absolute optical properties is often summarized as measuring the slope (versus source-detector distance; *ρ*) of a modified intensity amplitude and phase (i.e., modified complex reflectance). The chief complication of this technique is calibration, since each source-detector pair may have differing instrumental contributions (because of the individual source emission and detector sensitivity properties) and differing coupling factors between source/detector and sample. A method which compensates for the differing instrumental contributions is the so-called Multi-Distance (MD) scanning, where either the source or the detector is moved across the surface of the optical medium [22]. In doing so, the instrumental factors remain the same for each distance (same source and same detector used for each distance); thus, the measurement of slope is independent of these factors. However, differing coupling factors may still be present if during the scan the source (or detector) moves away or toward the tissue surface (in a non-contact case), or experiences a variable contact pressure (in a contact case). Aside from MD scanning, a potentially more effective and more easily implemented calibration free method may be employed. This method is the Self-Calibrating (SC) method, where a symmetric optical probe that features two sources and two detectors is used such that instrumental and coupling factors cancel in the calculation of slopes [23]. This method is also effective at suppressing motion artifacts [24] and allows calibration-free saturation measurements with Continuous-Wave (CW) methods after assuming the wavelength dependence of scattering [25]. The original purpose of the SC method was to achieve measurements of absolute optical properties with FD-NIRS. However, recently the optode geometry inspired by the SC method has been extended to the use of only the amplitude or phase of the complex reflectance in FD-NIRS [26,27]. This extension was named Dual-Slope (DS) since it relies on the average of two slopes measured with this special optode configuration.

One of the limitations of time-resolved NIRS methods, such as FD-NIRS, is the added instrumental complexity compared to CW. There has been work in broadband time-resolved spectroscopy [28]; however, such instruments require complex instruments (super-continuum lasers, single photon counting, and instrument response calibration). Because of this, the norm in time-resolved NIRS methods is the use of two or a few wavelengths as opposed to a continuous broadband spectrum [1]. In contrast, CW methods may use White-Light or broadband light sources, such as halogen lamps, and spectrometer detectors, since there is no need for measurements of fast temporal characteristics. These spectroscopic methods in CW are so-called Diffuse Reflectance Spectroscopy (DRS) due to their collection geometry and use of a spectrometer as a detector (instead of avalanche photodiodes or photomultiplier tubes typical in time-resolved methods). Therefore, CW broadband DRS (CW-bDRS) has the advantage of collecting data over many wavelengths but the disadvantage that absorption and scattering are coupled and inseparable due to the use of CW illumination. A solution that has become more and more common is to combine a time-resolved NIRS instrument at few wavelengths with a CW-bDRS system [29–34]. Such a technique allows extrapolation of scattering from few to many wavelengths, by assuming a power law decay of the reduced scattering coefficient with wavelength [2], as well as decoupling of absorption from scattering in the CW-bDRS data.

At this point, a small note on nomenclature is valuable. The acronym MD FD-NIRS refers to a frequency-domain method capable of measuring absolute optical properties (absorption and scattering) at discrete wavelengths from measurements at multiple distances between source and detector. The acronym CW-bDRS refers to a continuous-wave technique capable of measuring wavelength-resolved data (which depends on absorption and scattering) over a range of many wavelengths. Further, to achieve a calibration free full spectral technique, DS CW-bDRS is introduced. It is noted that the distinction between SC (self-calibrating) and DS (dual-slope) is the use of complex reflectance in SC FD-NIRS versus continuous-wave reflectance in DS CW-bDRS, for example. The main focus of this work will be to develop the DS CW-bDRS technique in combination with MD FD-NIRS to decouple absorption and scattering contributions to the absorbance spectra.

In this paper, a new DS CW-bDRS/MD FD-NIRS instrument is described and validated by measuring absolute absorption spectra of highly scattering media, or phantoms. The novelty of this work is the design of a CW instrument for broadband spectroscopy that implements DS methods for robust and calibration free measurements of absorbance spectra. The addition of MD FD-NIRS measurements at two wavelengths allows for the translation of absorbance spectra into quantitative absorption spectra. The organization of the paper is as follows. The methods, in Section 2, are split into techniques, in Section 2.1, where FD-NIRS and CW-bDRS are described, and is followed by experiments, Section 2.2, which lays out a phantom experiment, and then by an analysis, Section 2.3, which explains methods to calculate optical properties. Next, the results, Section 3, report measured phantom absorption spectra. Finally, the discussion, Section 4, elaborates on the validation of the DS CW-bDRS/MD FD-NIRS instrument.

## 2. Materials and Methods

### 2.1. Techniques

#### 2.1.1. Frequency-Domain Near-Infrared Spectroscopy

Frequency-Domain Near-Infrared Spectroscopy (FD-NIRS) was implemented with the purpose of measuring absolute optical properties of highly scattering media. Those being the absorption coefficient (*μ*_*a*_) and the reduced scattering coefficient $\left(\mu_{s}^{\prime }\right)$. A commercial FD-NIRS instrument was used (Imagent V2, ISS, Champaign, IL, USA) operating with a modulation frequency of 140.625 MHz and optical wavelengths of 690 and 830 nm.

**Figure 1.**
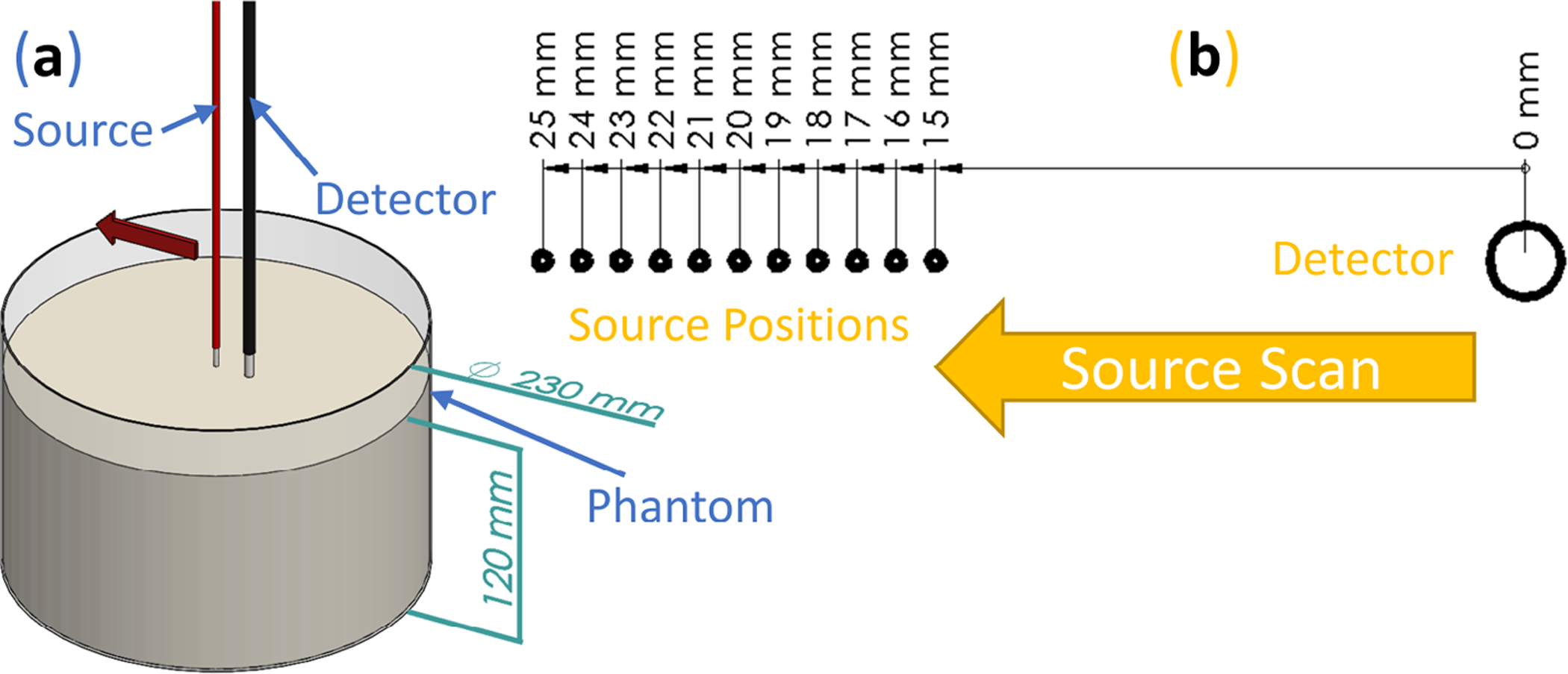
Frequency-domain near-infrared spectroscopy methods to achieve measurements of absolute optical properties. (**a**) Render of multi-distance scan done on diffuse optical phantoms. (**b**) Schematic of eleven different source positions realized during the multi-distance scan.

FD-NIRS was implemented in a Multi-Distance scan (MD FD-NIRS). To achieve this, a single detector fiber bundle (⌀3 mm) was held at a fixed location and two co-localized source fibers (⌀600 μm; two wavelengths) were scanned via a linear stage (Figure 1a). In doing so, the complex reflectance (amplitude and phase) was measured at eleven distances (from 15 to 25 mm spaced by 1 mm; Figure 1b). This MD scan allowed for measurements of complex reflectance slopes (amplitude and phase) versus source-detector distance without the need for calibration (assuming unchanging coupling with the sample since the same fibers are used for each distance and the fiber/sample contact remains about the same during the linear scan). These measurements were used to calculate the absolute optical properties of the diffuse medium as described in Section 2.3.

#### 2.1.2. Broadband Diffuse Reflectance Spectroscopy

**Figure 2.**
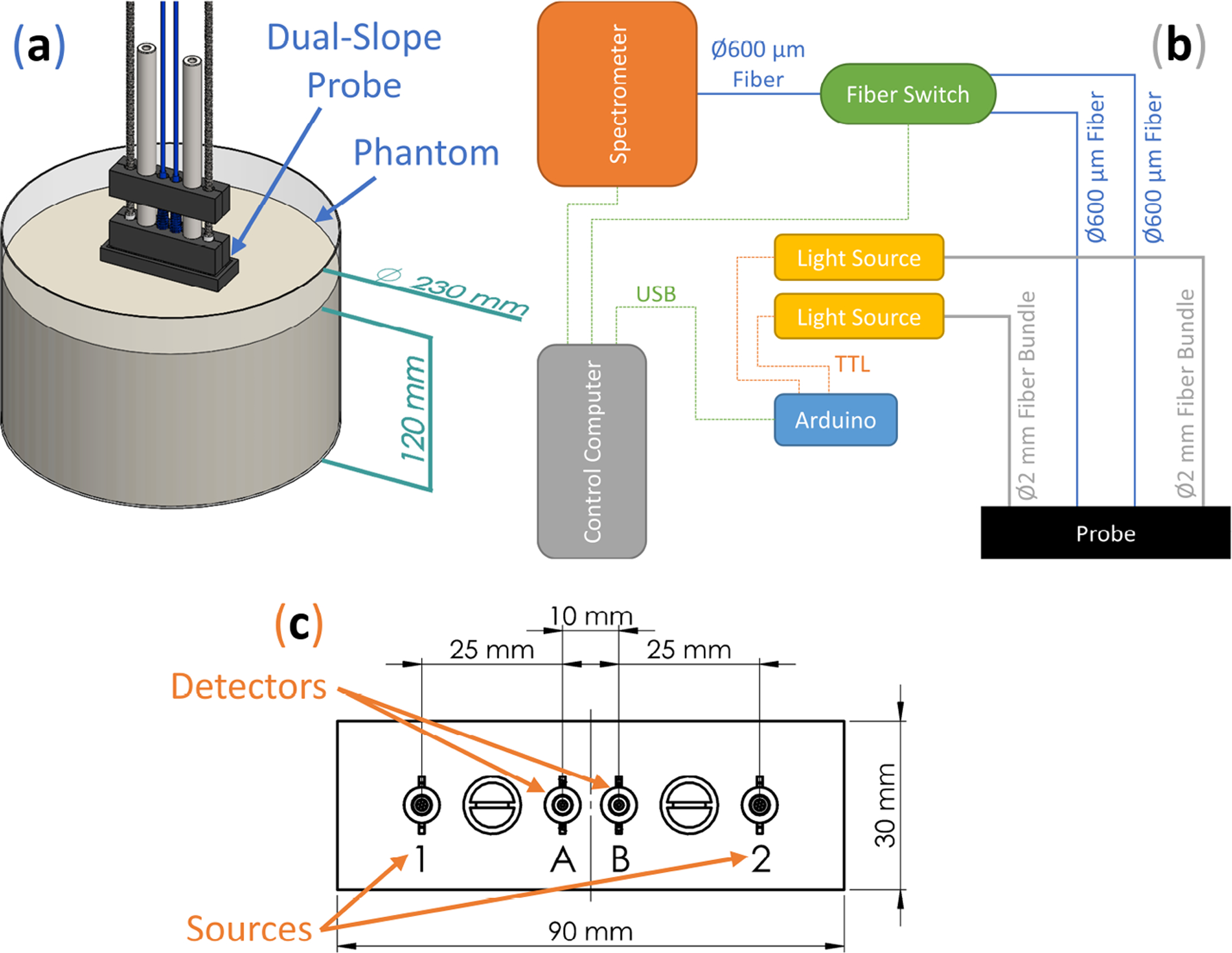
Broadband diffuse reflectance spectroscopy methods to achieve measurements of absolute absorption spectra. (**a**) Render of diffuse reflectance spectroscopy probe on a diffuse optical phantom. (**b**) Schematic of dual-slope diffuse reflectance spectroscopy device. Acronyms: Universal Serial Bus (USB) and Transistor-Transistor Logic (TTL). (**c**) Schematic of the source (1 and 2) and detector (A and B) positions on the dual-slope diffuse reflectance spectroscopy probe.

Continuous-Wave broadband Diffuse Reflectance Spectroscopy (CW-bDRS) was implemented with the purpose of measuring absolute absorption spectra of optically diffusive media (Figure 2a). This was realized using a Dual-Slope (DS) optode geometry which allowed for calibration free measurements of the CW reflectance slope (versus source-detector distance). The DS optode configuration (Figure 2c) used was the same as described previously [26]; it contains two source positions and two detector positions. The linearly symmetric arrangement resulted in a calibrated measurement of the slope (versus source-detector distance) of CW reflectance from 25 to 35 mm.

The DS CW-bDRS system was custom built to achieve the multiplexing needed for DS measurements. This measurement requires the reflectance signal be acquired from all combinations of sources (named 1 and 2) and detectors (named A and B; Figure 2c). To do so, both the sources and detectors must be multiplexed (Figure 2b). Sources were multiplexed by using two shuttered light sources (AvaLight-HAL-S-Mini, Avantes, Louisville, CO, USA), each connected to one source position. Shutter state was controlled via a Transistor-Transistor Logic (TTL) signal from a micro-controller (Uno R3, Arduino, Ivrea, Italy) which was connected to the control computer via Universal Serial Bus (USB). Light was delivered from the sources to the probe using ⌀2 mm fiber bundles. The light sources output an approximate black-body spectrum with a temperature of about 2650 K (peak at about 1000 nm). On the detector side, diffuse CW light was collected using a ⌀600 μm fiber at each of the two detector positions. Multiplexing of the detector positions was done using a 1 × 2 fiber switch (LBMB, Photonwares, Woburn, MA, USA) where the common end was connected to a spectrometer (AvaSpec-HERO, Avantes) via a ⌀600 μm fiber. The spectrometer was configured with a 500 μm slit and collected 1024 wavelengths between about 498 nm and about 1064 nm.

The bottleneck in the multiplexing sequence is the switching time of the fiber switch. Because of this, the cycle of source-detector position combinations was optimized to minimize fiber switch actuation. Naming source-detector combinations as source number (1 or 2), then detector letter (A or B; Figure 2c) as an example measurement sequence could be:

…1A]→[1A⇨2A▶2B⇨1B]→[1B⇨2B▶2A⇨1A]→[1A…

where “⇨” is a source switch, “▶” is a detector switch, and the square brackets show one full DS set acquisition. The reflectance from each source-detector combination is linearly interpolated to the average absolute time for their corresponding DS set. The four reflectance measurements that contribute to a DS measurement are synchronous, thus preventing potential temporal artifacts when studying dynamic samples or biological tissue. The specific acquisition parameters (including sampling rate and wavelength range considered) are stated in Section 2.2, and the analysis of the DS data (slope of CW reflectance versus source-detector distance) to yield absolute absorption spectra is described in Section 2.3.

### 2.2. Experiment

The purpose of the experiment was to validate the Dual-Slope broadband Diffuse Reflectance Spectroscopy (DS CW-bDRS) instrument. This was done using a liquid optical phantom which was measured at different dyes concentrations to confirm the DS CW-bDRS’s ability to distinguish spectral features and measure absolute absorption. A total of 5 L of the phantom was made and placed in a cylindrical tank (Figures 1a and 2a). The base of the phantom was made of 2% (reduced fat) Milk and Water (MW; 43% milk and 57% water volume fraction) such that the scattering properties were similar to those of biological tissue in the near-infrared. The scattering was assumed to not change as dyes were added since the addition of the dyes is expected to have a negligible effect on scattering. Each time the phantom was measured, the measurement was done by both Multi-Distance Frequency-Domain Near-Infrared Spectroscopy (MD FD-NIRS) and DS CW-bDRS.

**Figure 3.**
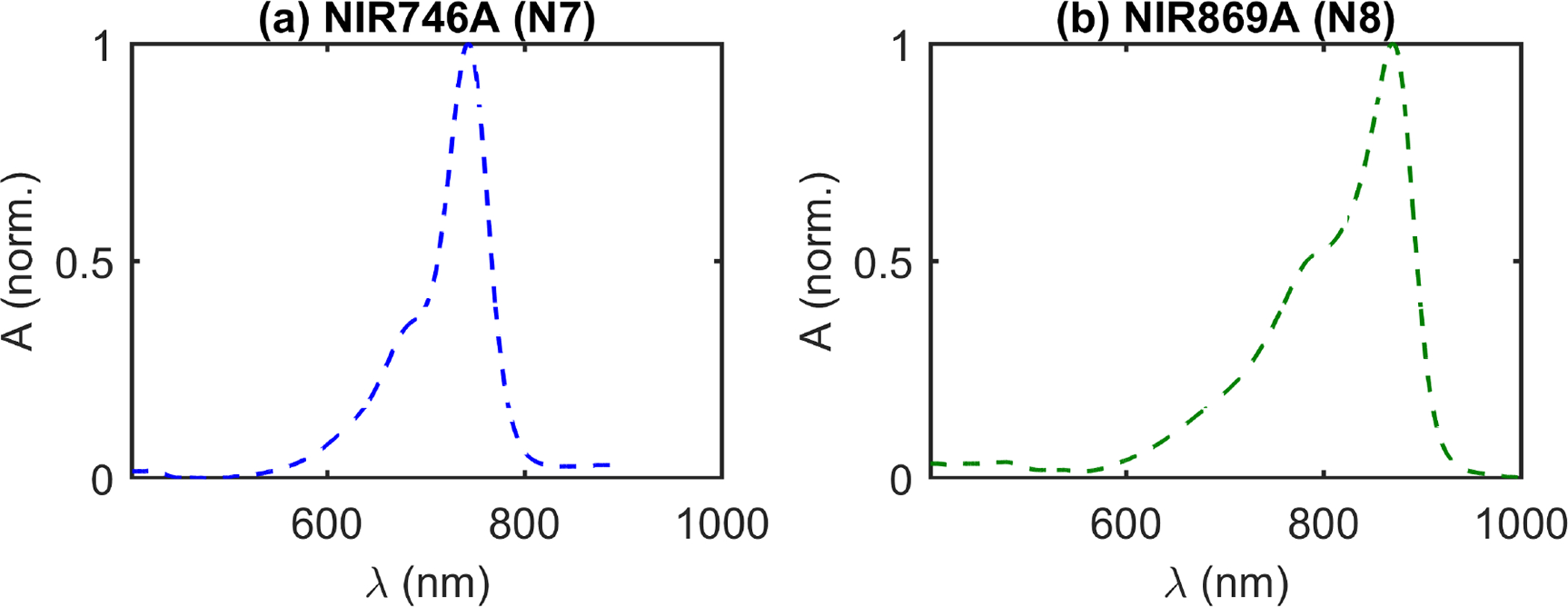
Expected spectral features of near-infrared dyes in water, provided by the manufacturer (QCR Solutions, Palm City, FL, USA). [37] (**a**) Normalized Absorbance (A) versus wavelength (*λ*) of NIR746A dye (N7). (**b**) Normalized A of NIR869A dye (N8).

Three dyes were added in the following order: black India-Ink (II; Higgins, Leeds, MA, USA), NIR746A (N7; QCR Solutions, Palm City, FL, USA), and NIR869A (N8; QCR Solutions). II is expected to have a relatively flat or decreasing absorption spectrum with wavelength in the Near-Infrared (NIR) range [35,36]. By contrast, N7 and N8 are expected to have absorption peaks at around 746 nm and 869 nm, respectively (Figure 3). However, the actual peak wavelength may shift depending on the chemical properties of the solvent [37,38].

The measurements and dye additions proceeded as follows (measurements meaning both MD FD-NIRS and DS CW-bDRS). First, the MW base phantom was mixed, then measured. Then, II was mixed in and the phantom measured again. Next, N7 was mixed and again the phantom measured. Finally, N8 was also mixed into the phantom and the measurement was done for a final time. The amount of each addition of dye was such that all absorption spectra were expected to stay within typical absorption values of biological tissue (approximately 0.005 to 0.020 mm^−1^) [2].

The temporal sampling parameters of the DS CW-bDRS system were set to yield a relatively fast sampling rate while still measuring a relevant wavelength range. Along these lines, DS CW-bDRS data were analyzed between 600 nm and 900 nm and the sampling rate was set to 2.5 Hz. For DS CW-bDRS 5 min of data were averaged for analysis. The MD FD-NIRS measurement took 30 s at each step for a total measurement time of 5.5 min (eleven steps; Section 2.1.1), and had a sampling rate of 2.8 Hz. These parameters were chosen such that the rate and time of DS CW-bDRS and MD FD-NIRS were approximately equal.

This phantom experiment resulted in MD FD-NIRS measurements at two wavelengths and DS CW-bDRS measurements between 600 nm and 900 nm for four phantoms (MW, MW+II, MW+II+N7, and MW+II+N7+N8). Section 2.3 describes the analysis of these data to result in absolute optical properties at two wavelengths and absolute absorption spectra.

### 2.3. Analysis

In Section 2.1, two techniques were described: Multi-Distance Frequency-Domain Near-Infrared Spectroscopy (MD FD-NIRS; Section 2.1.1) and Dual-Slope Continuous-Wave broadband Diffuse Reflectance Spectroscopy (DS CW-bDRS; Section 2.1.2). MD yields the spatial dependence of reflectance across multiple distances, while DS yields two symmetric spatial dependencies of reflectance. Additionally, FD-NIRS yields complex reflectance (at a few wavelengths), while CW-bDRS yields CW reflectance (at many wavelengths). However, regardless of these differences, the same basic analysis was used to find absolute optical properties (with the process repeated for each separate wavelength). In the following, we first describe the analysis of complex reflectance across multiple distances (MD FD-NIRS). Then, we explain how the method is modified to handle DS data and what additional assumptions are needed to use CW data (DS CW-bDRS).

Considering a semi-infinite medium with extrapolated-boundary conditions, the complex reflectance $\left(\tilde{R}\right)$, defined as the net optical flux exiting the tissue per unit source power, may be written as [2,39]:

(1) $$\tilde{R}=\frac{1}{4\pi }\left(\tilde{C_{1}}e^{-\tilde{\mu_{eff}}r_{1}}+\tilde{C_{2}}e^{-\tilde{\mu_{eff}}r_{2}}\right),$$

(2) $$\tilde{C_{1}}=z_{0}\left(\frac{1+\tilde{\mu_{eff}}r_{1}}{r_{1}^{3}}\right)\;\tilde{C_{2}}=-z_{0}^{\prime }\left(\frac{1+\tilde{\mu_{eff}}r_{2}}{r_{2}^{3}}\right),$$

(3) $$r_{1}=\sqrt{\rho^{2}+z_{0}^{2}}\;r_{2}=\sqrt{\rho^{2}+z_{0}^{\prime }{}^{2}},$$

where *z*_0_ is the depth of the real isotropic source $\left(z_{0}=1/\mu_{s}^{\prime }\right)$, $z_{0}^{\prime }$ is the height of the imaginary isotropic source $\left(z_{0}^{\prime }=-z_{0}+2z_{b}\right)$, *ρ* is the source-detector distance on the surface of the semi-infinite medium, and $\tilde{\mu_{eff}}$ is the complex effective attenuation coefficient. The extrapolated boundary height $\left(z_{b}=2A/\left(3\left(\mu_{s}^{\prime }+\mu_{a}\right)\right)\right)$ and the complex effective attenuation coefficient $\left(\tilde{\mu_{eff}}=\sqrt{3\left(\mu_{s}^{\prime }+\mu_{a}\right)\left(\mu_{a}-\omega n_{in}i/c\right)}\right)$ are both expressed in terms of the absorption coefficient (*μ*_*a*_), reduced scattering coefficient $\left(\mu_{s}^{\prime }\right)$. The remaining parameters are the angular modulation frequency (*ω*), the reflection parameter (*A*), the index of refraction inside the medium (*n*_*in*_; assumed to be 1.4, typical for biological tissue [2]), and the speed of light in vacuum (*c*). Additionally, *ω* = 2*π* *f*_*mod*_, where *f*_*mod*_ is the modulation frequency. Finally, *A* is a function of *n*_*in*_ and the index of refraction outside the medium (*n*_*out*_; assumed to be 1.0) [39]. To yield a linear relationship, Equation (1) can be rewritten as:

(4) $$\text{ln}\left[\frac{4\pi \tilde{R}}{\tilde{C_{1}}+\tilde{C_{2}}e^{\tilde{\mu_{eff}}\left(r_{1}-r_{2}\right)}}\right]=-\tilde{\mu_{eff}}r_{1},$$

which shows a linear dependence of a modified complex reflectance $\left(\tilde{y}=\text{ln}[4\pi \tilde{R}/\left(\tilde{C_{1}}+\tilde{C_{2}}e^{\tilde{\mu_{eff}}\left(r_{1}-r_{2}\right)}\right)]\right)$ on *r*_1_ (i.e., $\tilde{y}=-\tilde{\mu_{eff}}r_{1}$).

The linear complex slope $\left(-\tilde{\mu_{eff}}\right)$ in Equation (4) is used to calculate the desired optical properties (*μ*_*a*_ and $\mu_{\text{s}}^{\prime }$). However, $\tilde{y}$ is dependent on *μ*_*a*_ and $\mu_{\text{s}}^{\prime }$, and *r*_1_ is dependent on $\mu_{\text{s}}^{\prime }$. Thus, an iterative method is used to recover the $\tilde{\mu_{eff}}$:

(5) $$\tilde{y}\left[\mu_{a,k},\mu_{s,k}^{\prime }\right]=-{\tilde{\mu_{eff}}}_{k+1}r_{1}\left[\mu_{s,k}^{\prime }\right]+{\tilde{b}}_{k+1},$$

where *k* is the iteration number, and $\tilde{b}$ is the complex intercept (dependent on instrumental factors not considered in Equation (4)). For the iterative method, an initial guess of *μ*_*a*_ and $\mu_{\text{s}}^{\prime }$ is needed for *k* = 0. This guess was determined based on the linear slopes of complex reflectance amplitude and phase as previously described [19,21]. At each iteration, the current values of $\tilde{y}$ and *r*_1_ were used to fit a complex linear slope $\left(-\tilde{\mu_{eff}}\right)$:

(6) $${\left[\begin{array}{c} -\tilde{\mu_{eff}}\\ \tilde{b} \end{array}\right]}_{k+1}={\left[\begin{array}{cc} r_{1,1} & 1\\ \vdots & \vdots \\ r_{1,n} & 1 \end{array}\right]}_{k}^{+}{\left[\begin{array}{c} {\tilde{y}}_{1}\\ \vdots \\ {\tilde{y}}_{n} \end{array}\right]}_{k},$$

where the + superscript is the Moore-Penrose inverse, $\tilde{b}$ is the complex intercept (ignored), and there are *n* measurements (distances). In the case of MD FD-NIRS, *n* = 11 since there were eleven measurements of complex reflectance at eleven different source-detector distances. To convert $\tilde{\mu_{eff}}$ to *μ*_*a*_ and $\mu_{s}^{\prime }$ the following expressions were used:

(7) $$\mu_{t}^{\prime }=\frac{-2cℜ\left[\tilde{\mu_{eff}}\right]ℑ\left[\tilde{\mu_{eff}}\right]}{3n\omega },$$

(8) $$\mu_{a}=\frac{ℜ{\left[\tilde{\mu_{eff}}\right]}^{2}-ℑ{\left[\tilde{\mu_{eff}}\right]}^{2}}{3\mu_{t}^{\prime }},$$

where $\mu_{t}^{\prime }$ is the total reduced attenuation coefficient, and $\mu_{s}^{\prime }=\mu_{t}^{\prime }-\mu_{a}$. At each iteration, the new fitted $-\tilde{\mu_{eff}}$ yielded the *μ*_*a*_ and $\mu_{s}^{\prime }$ used to calculate $\tilde{y}$ and *r*_1_ for the next iteration (Equation (5)). The procedure terminated when the condition $\left|{\tilde{\mu_{eff}}}_{k}-{\tilde{\mu_{eff}}}_{k-1}\right|<10^{-4}\text{ L}/\text{mm}$ was met.

The above expressions show how MD FD-NIRS data were used to find absolute *μ*_*a*_ and $\mu_{s}^{\prime }$ at two wavelengths. To extend this to DS CW-bDRS, first, Equation (6) was modified to use DS data. DS yields two symmetric measurements of *R* versus *ρ* (where *R* is now real not complex since CW data is used). Thus, instead of Equation (6), the following was used for DS CW-bDRS:

(9) $$\mu_{eff_{k+1}}=\frac{-1}{2}{\left(\frac{y_{2,1}-y_{1,1}}{r_{1,2,1}-r_{1,1,1}}+\frac{y_{2,2}-y_{1,2}}{r_{1,2,2}-r_{1,1,2}}\right)}_{k},$$

where the first subscript of *y* and second subscript of *r*_1_ corresponds to source-detector distance, and the second and third subscript, respectively, corresponds to the symmetric slope in the DS set. This expression is simply the average of the slopes of *y* versus *r*_1_ between the two DS symmetric sets. In addition, note that the tildes were removed since *y* is now real (from real *R*). This results in a real effective attenuation coefficient $\left(\mu_{eff}=\sqrt{3\mu_{a}\left(\mu_{s}^{\prime }+\mu_{a}\right)}\right)$ instead of a complex one. Equations (1)–(5) may all be used as is, with removing the tilde hats to now represent real data.

Since the DS CW-bDRS method yields a real *μ*_*eff*_, $\mu_{s}^{\prime }$ must be known to find *μ*_*a*_ using the following expression instead of Equation (8):

(10) $$\mu_{a}=\sqrt{\frac{\mu_{s}^{\prime 2}}{4}+\frac{\mu_{eff}^{2}}{3}}-\frac{\mu_{s}^{\prime }}{2}.$$

To address the need to know $\mu_{s}^{\prime }$, the data from the MD FD-NIRS measurements were used. FD-NIRS yielded a measurement of $\mu_{s}^{\prime }$ at two wavelengths ($\mu_{s,FD}^{\prime }\left(\lambda_{1}\right)$ and $\mu_{s,FD}^{\prime }\left(\lambda_{2}\right)$; where *λ* is wavelength). These $\mu_{s}^{\prime }$ measurements are then extrapolated using the following expression to find $\mu_{s}^{\prime }$ at all the wavelengths used by bDRS:

(11) $$\mu_{s}^{\prime }(\lambda )=\mu_{s,FD}^{\prime }\left(\lambda_{1}\right){\left(\frac{\lambda }{\lambda_{1}}\right)}^{\frac{-\text{ln}\left[\mu_{s,FD}^{\prime }\left(\lambda_{2}\right)/\mu_{s,FD}^{\prime }\left(\lambda_{1}\right)\right]}{\text{ln}\left[\lambda_{1}/\lambda_{2}\right]}}.$$

Therefore, when analyzing DS CW-bDRS data, *μ*_*a*_ is found for each wavelength using the assumed $\mu_{s}^{\prime }$ expressed above for each wavelength.

In summary, the same basic method, based on iteratively fitting to Equation (4), is used for MD FD-NIRS and DS CW-bDRS to find absolute optical properties. DS differs from MD in the calculation of slope, and the $\mu_{s}^{\prime }$ from MD FD-NIRS is extrapolated to find an assumed $\mu_{s}^{\prime }$ for each DS CW-bDRS wavelength.

## 3. Results

The phantom experiment described in Section 2.2 consisted of measuring four phantoms, each with both Multi-Distance Frequency-Domain Near-Infrared Spectroscopy (MD FD-NIRS; Figure 1a) and Dual-Slope Continuous Wave broadband Diffuse Reflectance Spectroscopy (DS CW-bDRS; Figure 2a). The phantoms were Milk and Water (MW), that with India Ink added (MW+II), then that again with NIR746A added (MW+II+N7), and then that yet again with NIR869A added (MW+II+N7+N8).

**Figure 4.**
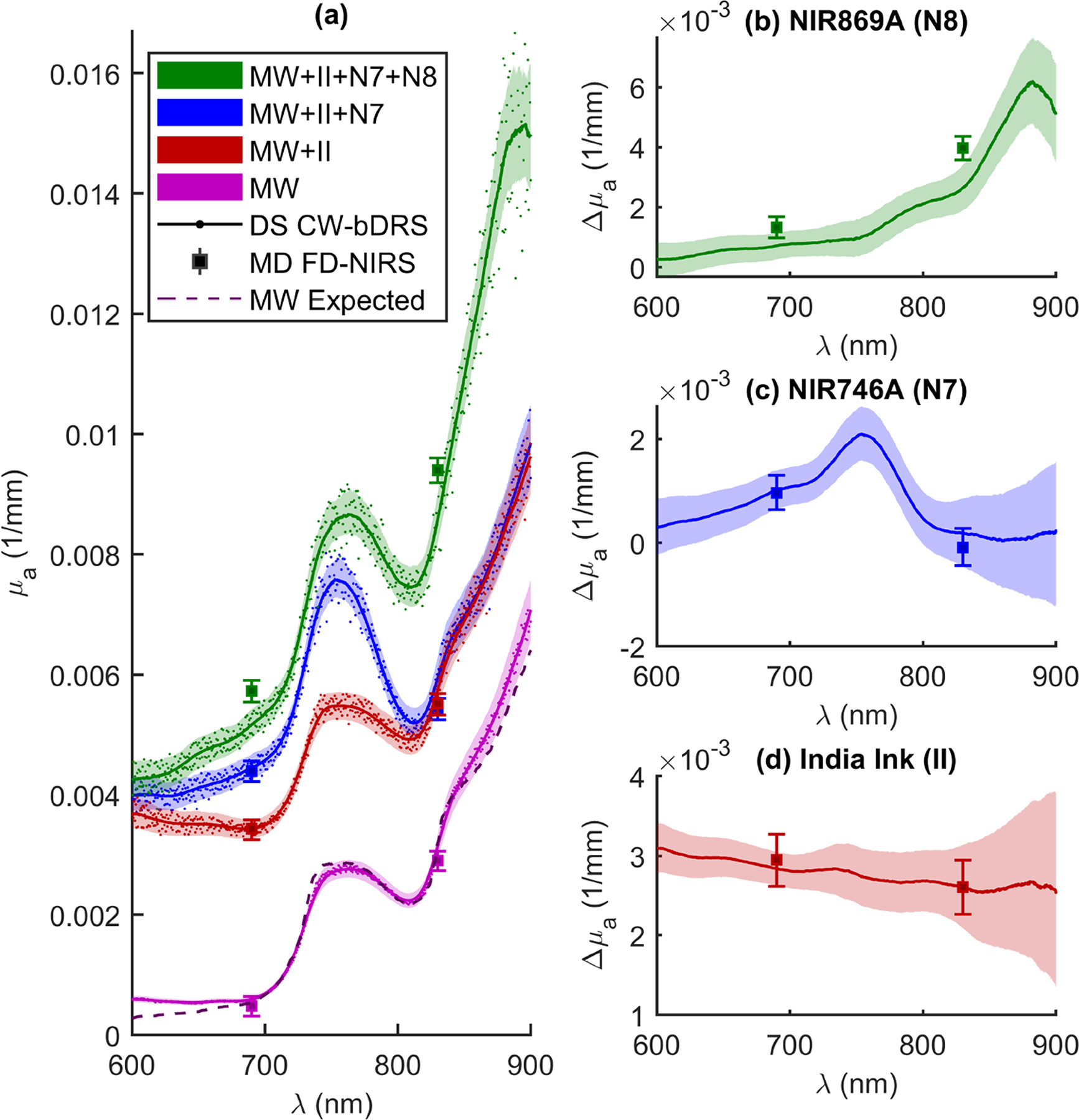
Results from phantom experiment. (**a**) Absolute absorption (*μ*_*a*_) spectra as a function of wavelength (*λ*). Showing results from Dual-Slope Continuous-Wave broadband Diffuse Reflectance Spectroscopy (DS CW-bDRS) and Multi-Distance Frequency-Domain Near-Infrared Spectroscopy (MD FD-NIRS) measurements. Spectra shown for the following phantoms: Milk and Water (MW), MW plus India Ink (II), MW plus II plus NIR746A (N7), and, finally, MW plus II plus N7 plus NIR869A (N8). DS CW-bDRS points show individual wavelength measurements and lines show smoothed (moving average) spectra for visualization. Dashed line shows the expected spectrum for MW modeled as water and lipid. (**b**) Change in absorption (Δ*μ*_*a*_) from adding N8 (i.e., $\mu_{a}^{MW+II+N7+N8}-\mu_{a}^{MW+II+N7}$), (**c**) Δ*μ*_*a*_ from adding N7 (i.e., $\mu_{a}^{MW+N7}-\mu_{a}^{MW+II}$), and (**d**) Δ*μ*_*a*_ from adding II (i.e., $\mu_{a}^{MW+II}-\mu_{a}^{MW}$).

Figure 4 shows the results of this experiment. The absolute absorption (*μ*_*a*_) spectrum is shown across wavelengths (*λ*) for all four phantoms and two measurement methods (Figure 4a). Additionally, Figure 4a shows a dashed line representing the modeled (as 99.1% water [40,41] and 0.9% lipid [42] volume fractions) absorption of the MW phantom. The coefficient of determination (*R*^2^) was calculated for the MW data and model, yielding a value of 0.98, implying that 98% of the variance in the MW data is explained by the modeled MW absorption. In Figure 4b–d, the change in absorption (Δ*μ*_*a*_) as a result of adding the three different dyes (II, N7, and N8) is shown. The error regions in all plots are dominated by the systematic uncertainty in the distances on the DS-CW-bDRS probe (estimated at ±0.1 mm for chained dimensions) and within the MD-FD-NIRS measurement (estimated at ±0.5 mm for initial position and ±0.01 mm for scan pitch). Note the flat absorption contribution from II and the spectral features from N7 and N8. Particularly, a peak between 700 nm and 800 nm for N7 and a peak between 800 nm and 900 nm for N8. How these results serve to validate the instrument will be discussed in Section 4. Briefly, notice the agreement between the MD FD-NIRS and DS CW-bDRS measurements and the agreement between the expected MW spectrum and the DS CW-bDRS measured MW spectrum.

## 4. Discussion

The experiment involved a liquid phantom and sought to validate the Dual-Slope Continuous Wave broadband Diffuse Reflectance Spectroscopy (DS CW-bDRS) instrument (Sections 2.2 and 3). This was done by measuring a highly scattering phantom as dyes were progressively added (Figure 4). The expectation was that the DS CW-bDRS (in combination with Multi-Distance Frequency-Domain Near-Infrared Spectroscopy; MD FD-NIRS) instrument would be able to measure the known spectral features of the dyes and the absolute absorption of the phantom.

First, before any dyes were added (just Milk and Water; MW), the phantom was measured to yield an absorption spectrum dominated by water (Figure 4a). This MW spectra had the expected spectral features of water (hump at about 750 nm and sharp increase starting at about 800 nm) [41]. Further, the expected MW spectrum agrees (within error) with the experimental data for the spectral range between 690 nm and 830 nm, where the reduced scattering coefficient is interpolated (not extrapolated) from the MD FD-NIRS measurement. Below 690 nm, the agreement is lost likely due to the very low water absorption, such that, even low absorption, contributions from fat, proteins, or other milk constituents in the MW medium may become detectable. Above 830 nm, the agreement also degrades possibly because of incorrect reduced scattering values (from extrapolation) or contributions from other absorbers in the milk other than water. Overall, the quantitative agreement between the expected and measured MW spectra are quite good with a coefficient of determination (*R*^2^) of 0.98, indicating an accurate measurement of absolute absorption by DS CW-bDRS.

Further, the absorption measured by MD FD-NIRS agrees within error with DS CW-bDRS at all points of comparison except for NIR869A (N8). For N8, the difference between the measurements is about 10%, and the combined errors for the two measurements amount to about 8% (dominated by uncertainty in the distances; Section 3). Admittedly, the reduced scattering found by MD FD-NIRS is used to find the absorption for DS CW-bDRS; thus, the measurements are related (Section 2.3). However, the observed agreement demonstrates a reliable absolute measurement of the slope of diffuse reflectance with the DS CW-bDRS instrument, given the accurate measurements with the scanning MD method used by MD FD-NIRS in controlled laboratory conditions. In the case of N8, where the two instruments do not agree, the most likely explanation is instrumental error (distance uncertainty and boundary conditions). The distance uncertainty accounts for most of the difference between the measurements, and the remaining unaccounted disagreement may be explained by uncertainties in boundary conditions (true depth of fibers in phantom, existence of meniscus, etc.). These distance uncertainties and boundary effects may have changed for each measurement, since after the addition of each dye the instruments needed to be re-setup and placed on the phantom. This may explain why instrumental errors impacted differently the measurements of different dyes. Additionally, there is evidence that the NIR dyes are not stable over time (discussed below), and the two measurements are not simultaneous (5–10 min between measurements). Any of these considerations may have led to the lack of agreement for N8. However, given the existence of these considerations and the close agreement in six out of eight cases, we find that the results serves to validate the absolute absorbance measured by DS CW-bDRS.

Focusing on the individual dye additions allows for validation of the measurement of spectral features. The first dye added was India Ink (II), which is expected to have a flat or decreasing spectral dependence with wavelength [35]. The change in the absorption spectrum observed confirmed this wavelength dependence and, again, agreed with the measurements at two wavelengths with MD FD-NIRS (Figure 4d). Further evaluation was carried out to estimate the recovered concentration of II given the measured change in absorption. The true concentration (in volume fraction) of II in the phantom was 5.7 × 10^−6^, given the phantom volume and the amount of ink added. The same II was measured in transmission in non-diffuse solutions of known concentrations to yield spectra of the total attenuation coefficient (*μ*_*t*_; assuming only unscattered light is detected). To yield the spectrum of absorption (*μ*_*a*_; needed to recover concentration in the diffuse experiment) for II, the single scattering albedo (*a*) must be assumed (*μ*_*a*_ = *μ*_*t*_(1 − *a*)). This must be considered for II since it is not in solution in water but instead a suspension of carbon particles. Wavelength independent values of *a* = 0 to *a* = 0.15 were assumed which yielded recovered concentrations of 5.2 × 10^−6^ to 6.1 × 10^−6^, respectively. This range of low albedo values of pure II indicates that its scattering coefficient is much smaller than the absorption coefficient, which is consistent with the literature [35,36]. The range resulted in measurement errors of −8.8% to 7.0% (given true value of 5.7 × 10^−6^). Therefore, it is expected that DS CW-bDRS is capable of recovering accurate chromophore concentrations given that the extinction spectra is known (not the case here since the albedo for II was not measured). For NIR746A (N7) and N8, the concentration recovery was not carried out due to temporal and spectral instability of the dyes (discussed below). Future experiments will be undertaken to validate the accurate chromophore concentration recovery of DS CW-bDRS using soluble and stable dyes.

**Figure 5.**
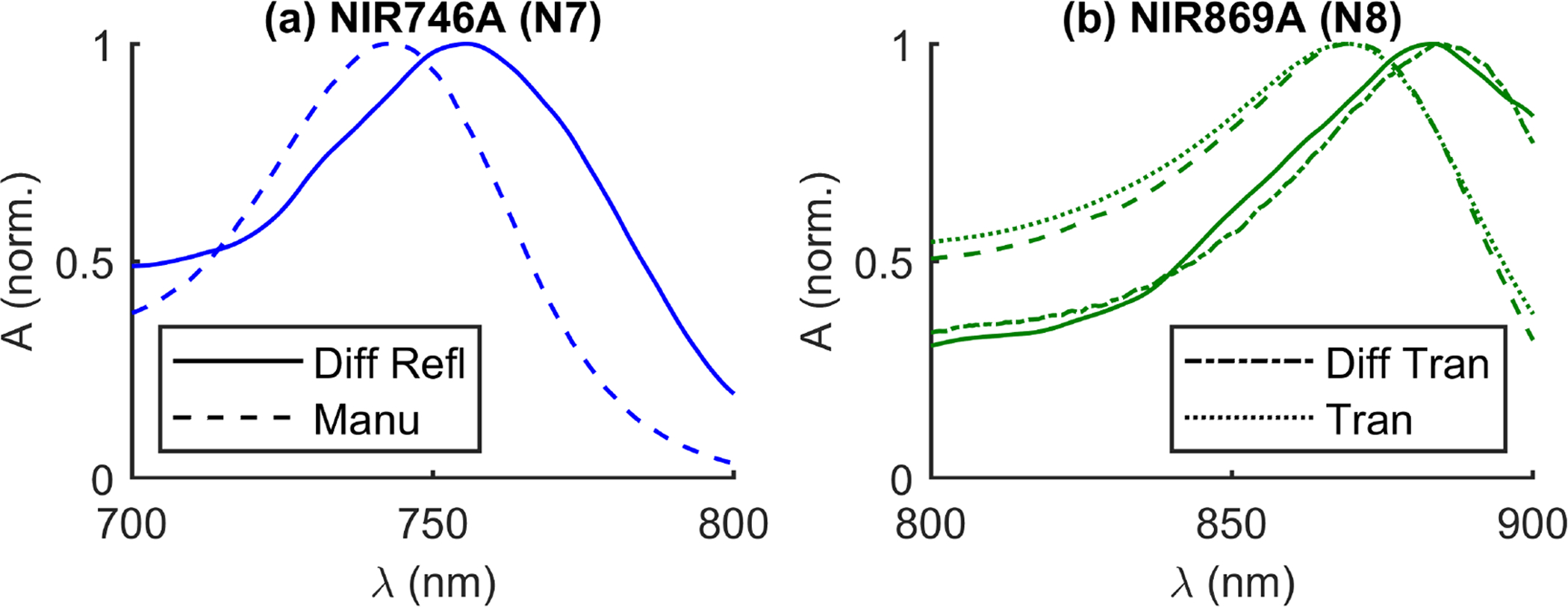
Comparison of wavelength (*λ*) normalized Absorbance (A) peak locations for two dyes. Four types of spectra shown: Diffuse Reflectance (Diff Refl, i.e., in milk), provided by the Manufacturer (Manu; (QCR Solutions, Palm City FL, USA) [37], i.e., in water), Diffuse Transmission (Diff Tran, i.e., in milk), and Transmission (Tran, i.e., in water). (**a**) NIR746A (N7) dye. (**b**) NIR869A (N8) dye.

Moving on from II, the next two dyes were expected to feature a more interesting spectrum (Figure 3). NIR746A (N7) was added after II and the change of absorption presented in Figure 4c. The expected peak between 700 nm and 800 nm was present. However, upon closer examination, the exact peak location is shifted about 12 nm higher for the DS CW-bDRS measurement compared to the expected spectrum (Figure 5a). When the next and final dye, NIR869A (N8), was added, yet again there was a peak present in approximately the expected location (Figure 4b). But, as with N7, there was an approximate 12-nm shift to longer wavelengths in the DS CW-bDRS measurement. This is consistent with a bathochromic shift, which is possible for these dyes given the manufacturer information and previous studies [37,38]. The hypothesis is that, when in milk, the dyes exhibit a bathochromic shift due to the different bulk polar nature of milk versus water. To test this possibility, two further measurements were done on N8. N8 was measured in transmission (in a semi-micro cuvette), both in water and in the MW mixture (Figure 5b). The results show that the transmission spectral absorbance for N8 in milk matches the DS CW-bDRS peak location, whereas the transmission spectral absorbance of N8 in water matches the manufacturer provided spectra. Thus, this transmission experiment supports the hypothesis that these dyes exhibit a bathochromic shift of about 12 nm when in the MW mixture. However, it is unknown if the amplitude of the absorption peak is also effected (hyperchromic or hypochromic shift) for these dyes. The dyes were also found to be temporally unstable, as extended time in solution caused N7 to lose its near-infrared absorption peak, and N8’s peak shifted roughly 200 nm to the blue. These spectral and temporal instabilities stopped the analysis of the concentrations of these dyes in the diffuse phantom. But, despite this, DS CW-bDRS was still capable of distinguishing the dye’s spectral features.

Summarizing the discussion above, these results on the liquid phantom serve to validate the DS CW-bDRS instrument’s ability to measure spectral features and absolute values of absorption in a diffuse medium. In six of eight cases (all except 690 nm and 830 nm for N8), the absolute absorption measured with DS CW-bDRS agreed within error with MD FD-NIRS. DS CW-bDRS also accurately measured the expected absorption spectrum of MW (*R*^2^ = 0.98). Additionally, the DS CW-bDRS instrument correctly measured the flat spectra of II and the peaks of N7 and N8. The concentration of II was estimated, suggesting the ability to recover chromophore concentrations given the extinction. Finally, it was confirmed that the recovered peak locations of the N7 and N8 dyes are what is expected for these dyes in milk. Future work will be done to validate the overall methods ability to accurately recover chromophore concentrations.

## 5. Conclusions

In this article, we have presented a new Dual-Slope Continuous Wave broadband Diffuse Reflectance Spectroscopy (DS CW-bDRS) instrument. Experiments on highly scattering liquid phantoms demonstrated the instrument’s capability of measuring absolute absorbance spectra without any need for instrumental calibration. By combining DS CW-bDRS and Frequency-Domain Near-Infrared Spectroscopy (FD-NIRS; to account for scattering contributions to the absorbance spectrum), we were able to measure absolute absorption spectra of the liquid phantoms that contained a combination of three dyes. These experiments demonstrated the technique’s ability to perform absolute spectral absorption measurements and retrieve the correct spectral features of various dyes. Future work will focus on further validation of chromophore concentration measurements. The importance of this work lies in the development of the DS CW-bDRS instrument. This DS CW-bDRS instrument is novel in that it combines DS and bDRS to achieve calibration-free measurements and provides a valuable tool for absolute spectral measurements of highly scattering media, including biological tissues.

## Data Availability

Data and supporting codes are available upon request.
